# The effect of pioneer carrion beetles on the emission of volatile organic compounds and carrion insect community assembly

**DOI:** 10.1002/ece3.10818

**Published:** 2023-12-12

**Authors:** Minobu Ito, Atsuko Nishigaki, Masami Hasegawa

**Affiliations:** ^1^ Department of Biology, Graduate School of Science Toho University Funabashi Japan; ^2^ Department of Environmental Science, Graduate School of Science Toho University Funabashi Japan

**Keywords:** carrion ecology, community assembly, ecological succession, processing chain, Silphinae, volatile organic compounds

## Abstract

Mechanisms of carrion insect succession have been interpreted separately from interspecific interactions between early and later colonists or from changes in volatile organic compounds perceived by insects resulting from the progression of decomposition. To link these perspectives, we examined through laboratory and field experiments whether the modification of volatile organic compounds by early colonists could be a mechanism of succession. In the laboratory experiment, we used *Necrophila japonica* (Coleoptera, Staphylinidae) as an early colonist and examined its effects on the emissions of important volatile attractants for carrion insects, dimethyl disulfide (DMDS) and dimethyl trisulfide (DMTS) from carcasses. We collected DMDS and DMTS, using the static headspace method, under the following conditions: (i) rat carcass, (ii) rat carcass with artificial damage to the abdomen, (iii) rat carcass fed on by 10 *Ne. japonica* individuals, and (iv) 10 *Ne. japonica* individuals, and analyzed the collected gases using a gas chromatograph‐mass spectrometer. After 12 and 30 h, carcasses fed on by *Ne. japonica* emitted higher concentrations of DMDS and DMTS than in other conditions. In the field experiment, we examined the effects of DMDS and DMTS on the attraction of carrion insects using traps baited with a mixture of DMDS and DMTS, hexane (odors unrelated to carcasses), or an empty microtube. Traps baited with DMDS and DMTS attracted more necrophagous species and individuals than traps not baited with this combination. These results showed that accelerated emissions of DMDS and DMTS from carcasses caused by early colonists may contribute to community assembly during carrion insect succession.

## Graphical abstract

We examined the effect of an early colonist, *Necrophila japonica* (Coleoptera, Staphylinidae), on the volatile organic compound emissions from carcasses and the effect of volatile organic compounds on carrion insect community assembly. We showed that carcasses fed on by *Ne. japonica* accelerated the emissions of dimethyl disulfide (DMDS) and dimethyl trisulfide (DMTS) and that DMDS and DMTS can contribute to attracting carrion insects. Therefore, our results suggest that modifications by early colonists that accelerated the emissions of DMDS and DMTS may promote community assembly during carrion insect succession.

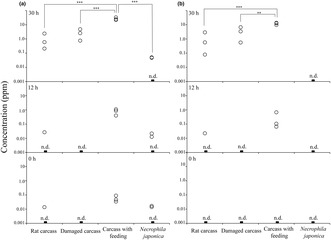

## 1 INTRODUCTION

Vertebrates create discrete and nutrient‐rich patches after they die (Carter et al., 2007; Finn, 2001), and various organisms, such as microbes, insects, and vertebrates, colonize and interact on their carcasses (Barton et al., 2013). Insects, particularly, colonize carcasses with relatively predictable sequences (Schoenly & Reid, 1987) and contribute significantly to decomposition (Parmenter & MacMahon, 2009; Payne, 1965; Simmons et al., 2010). Therefore, their colonization and successional patterns have been extensively studied in ecology and forensic entomology, which estimates postmortem intervals using carrion insects (Amendt et al., 2004; Goff, 1993). Flies and Silphini beetles (Staphylinidae, Silphinae) are considered the main colonists that interact on large carcasses (Anderson, 1982; Matuszewski & MĄdra‐Bielewicz, 2022), and *Nicrophorus* beetles (Staphylinidae, Silphinae, Nicrophorini) interact on small carcasses (Anderson, 1982; Trumbo & Bloch, 2002). However, a recent study has revealed interspecific interactions between the Silphini beetle, *Necrophila japonica*, and *Nicrophorus concolor* on small carcasses (Ito, 2022). In this study, carcasses fed on by *Ne. japonica* were colonized by *Ni. concolor* earlier than carcasses that were not fed upon by *Ne. japonica*. Therefore, *Ne. japonica*, an early colonist, promoted the colonization by *Ni. concolor*, a later colonist.

The mechanisms that cause insect colonization and succession on carcasses have traditionally been interpreted from a macroscopic perspective, such as interspecific interactions between insects. This idea is referred to as the facilitation (Connell & Slatyer, 1977) or processing chain model (Heard, 1994), and succession is considered to be driven by early colonists modifying carcasses to suitable conditions for later colonists to colonize or utilize. Although physical modifications, such as the penetration of epithelial tissue by ants and fly larvae that enable the utilization of internal tissue by later colonists (Braack, 1987; Eubanks et al., 2019; Schoenly & Reid, 1987), are regarded as modifications by early colonists, sufficient empirical studies on this phenomenon have not been conducted (Michaud et al., 2015; Nadeau et al., 2015).

However, recent studies have elucidated the mechanisms of carrion insect succession from a microscopic perspective. These studies have demonstrated temporal changes in carrion insects corresponding to changes in volatile organic compounds emitted from carcasses (Recinos‐Aguilar et al., 2020; Trumbo & Newton, 2022). This idea is called the gas‐emanation hypothesis (Nadeau et al., 2015), and carrion insect succession is thought to be driven by the temporal change in volatile organic compounds perceived by different insects (Dekeirsschieter et al., 2009, 2012; Forbes et al., 2014; Kasper et al., 2012; Stadler et al., 2013) resulting from microbial succession on carcasses (Pechal, Crippen, et al., 2014). In particular, sulfur‐containing volatile organic compounds are considered important attractants for major carrion insects, such as flies and carrion beetles (Verheggen et al., 2017).

Although the mechanisms of carrion insect succession have been independently interpreted from the macroscopic and microscopic perspectives, no studies have linked these perspectives. A recent study showed that *Nicrophorus* beetles modify volatile organic compounds emitted from carcasses (Trumbo et al., 2021). Therefore, if early colonists modify the emissions of volatile organic compounds perceived by later colonists, the gas‐emanation hypothesis may be incorporated into traditional models based on interspecific interactions between insects (i.e., facilitation and processing chain models). This integrated approach may explain the interspecific interactions between Silphini and *Nicrophorus* beetles (Ito, 2022) described above. Because some *Nicrophorus* species are attracted to carcasses by sulfur‐containing volatile organic compounds, such as dimethyl disulfide (DMDS) and dimethyl trisulfide (DMTS) (Kalinová et al., 2009; Podskalská et al., 2009; von Hoermann et al., 2016), the promotion of *Ni. concolor* colonization by *Ne. japonica* may be explained by the modification of DMDS and DMTS emissions by *Ne. japonica* feeding. In contrast, the regular gas‐emanation hypothesis may explain the *Ni. concolor* colonization of carcasses not fed on by *Ne. japonica*.

In this study, we aimed to clarify whether the modification of DMDS and DMTS by *Ne. japonica* could be one of the mechanisms of carrion insect succession. For this purpose, we compared the dynamics of DMDS and DMTS in carcasses with and without feeding by *Ne. japonica* in the laboratory. We hypothesized that carcasses fed on by *Ne. japonica* show accelerated emissions of DMDS and DMTS. In addition, we examined the role of DMDS and DMTS in attracting carrion insects, including *Ni. concolor*, by comparing the species and abundance captured between traps baited and not baited with DMDS and DMTS in the field. For the field experiment, we hypothesized that traps baited with DMDS and DMTS would attract more insects than traps not baited with DMDS and DMTS.

## 2 MATERIALS AND METHODS

### 2.1 Insect

For the laboratory experiment, wild *Ne*. (*Eusilpha*) *japonica* (Coleoptera, Staphylinidae, Silphinae) were captured in the northern part of Chiba Prefecture, eastern Japan (35°45′2″ N, 140°12′48″ E) in September 2013. Despite their flight ability, *Ne. japonica* adults are thought to search for food by walking (Nagano & Suzuki, 2003a), as they are not attracted to carrion hanging from trees or enclosed by plastic pipes (Ito, 2022; Nagano & Suzuki, 2003a). They can use both invertebrate (earthworm) and vertebrate carcasses for larval development and egg production (Watahiki & Sasakawa, 2019), often inhabiting them in high densities, reflecting their wide range of feeding habitats (Nagano & Suzuki, 2003a). Therefore, we directly captured adult *Ne. japonica* individuals walking on the forest floor. After collection, 11–13 adults were housed per container (width, 26.2 cm; depth, 19 cm; height, 10 cm) and kept in the laboratory at 24°C for approximately a day until the start of the experiment. Moistened tissue paper was placed in each container to allow the beetles to drink water ad libitum.

### 2.2 Chemicals

Commercial standards of dimethyl disulfide (99.0+%), dimethyl trisulfide (96.0+%), and hexane (96.0+%) were obtained from Wako Pure Chemicals (Osaka, Japan) and used for the following experiments.

### 2.3 Effect of *Ne. japonica* on DMDS and DMTS emissions from carcasses

**FIGURE 1:**
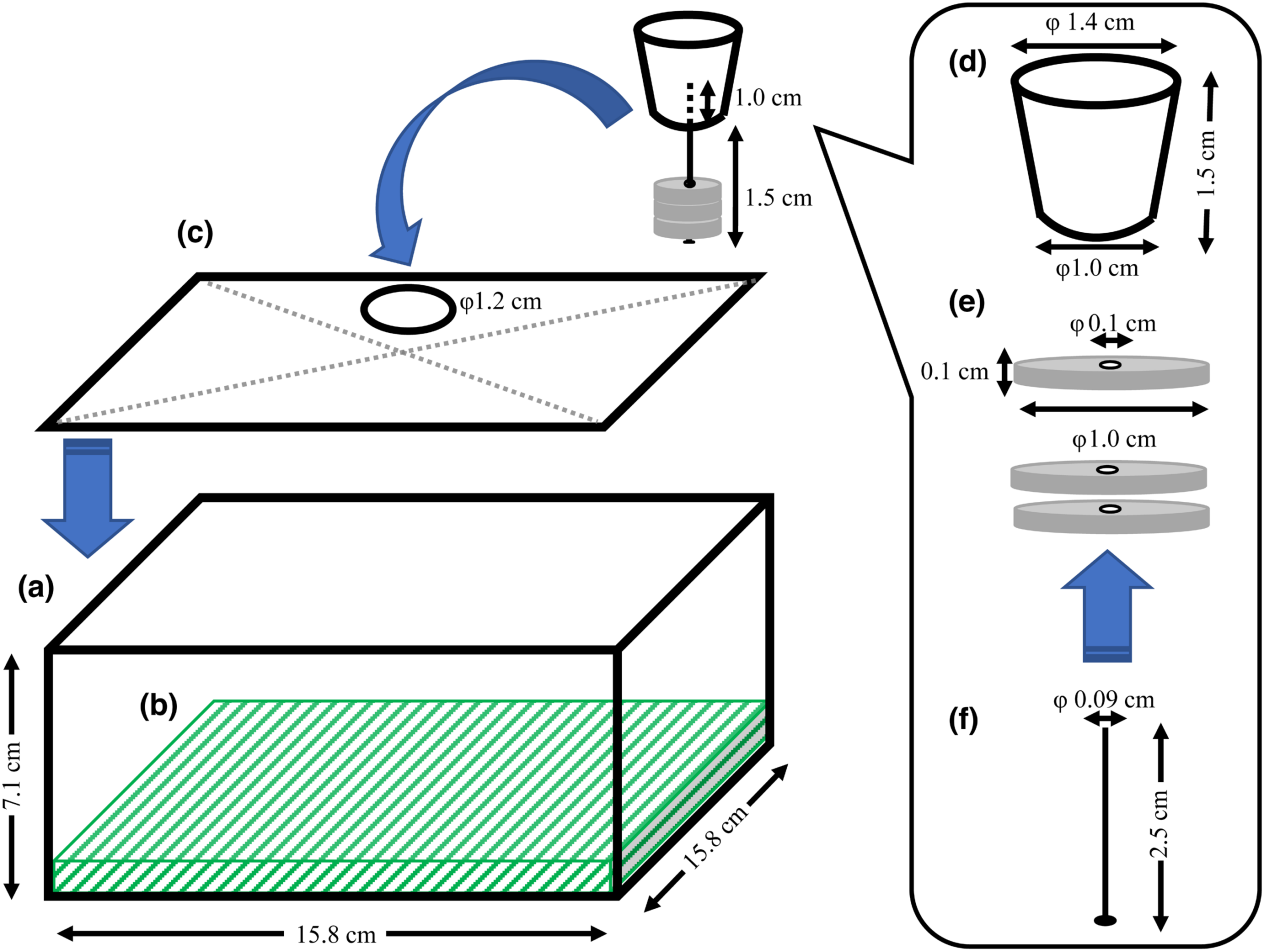
Schematic diagram of the experimental device and sampling method used to collect dimethyl disulfide (DMDS) and dimethyl trisulfide (DMTS). A rat carcass and/or 10 adults of *Necrophila japonica* were placed into a polypropylene container (a) with a plastic artificial lawn (b) at the beginning of trials. During the collection of DMDS and DMTS, the container was covered by a lid with silicone rubber packing and a 1.2 cm hole (c). Depending on the conditions and timing of collection, one or three adsorbents with 0.1 cm holes, MonoTrap DCC18 (e), were suspended at the headspace of the container using a rubber stopper (d) and a round head nail (f).

To evaluate the effects of *Ne. japonica* on DMDS and DMTS emissions from carcasses, a laboratory experiment was conducted in September 2013. As experimental devices, polypropylene containers (width, 15.8 cm; depth, 15.8 cm; height, 7.1 cm; volume, 1.05 L; TLS‐40, Asvel, Nara, Japan) each with a lid equipped with silicone rubber packing were used (Figure 1a). A 1.2‐cm hole was made in each of the lids, and a plastic artificial lawn was laid on the bottom of the containers (Figure 1b,c).

Frozen rat carcasses (mean = 39.9 g, SD = 3.0 g) were purchased from a pet store registered with the prefectural governor and used immediately after thawing in a water bath. By introducing rat carcasses and/or 10 adult *Ne. japonica* individuals into the containers, we created the following conditions: (i) A container containing only a rat carcass. This condition was used to reproduce DMDS and DMTS emissions during normal decomposition. (ii) A container with a rat carcass in which 2 cm squares of epithelial and connective tissue of the abdominal region of the carcass were cut off using flame‐sterilized dissecting scissors (damaged carcass). This condition was used to evaluate the effect of physical damage on DMDS and DMTS emissions. (iii) A container with a rat carcass and 10 adult *Ne. japonica* individuals (carcass with feeding). In this condition, *Ne. japonica* was allowed to feed on the carcass ad libitum during the experiment. (iv) A container containing only 10 adult *Ne. japonica* individuals (*Ne. japonica*). In this condition, *Ne. japonica* was kept without food to evaluate DMDS and DMTS originating from *Ne. japonica*. We also prepared a container without a rat carcass or *Ne. japonica* to evaluate the DMDS and DMTS originating from the experimental devices. A vial lid (diameter, 4.0 cm) filled with moistened tissue paper was placed in all containers to allow the beetles to drink water ad libitum. All containers were covered with a mesh screen and placed in a draft chamber maintained at 24°C and approximately 35% relative humidity (relative humidity was measured after the experiment) when not being sampled. All conditions were tested simultaneously in one trial, and the trials were repeated three times. Although one *Ne. japonica* died in two of the three trials under the “carcass with feeding” and “*Ne. japonica*” conditions during the trials, the dead individuals were removed, and the trials continued without replenishing the containers with new individuals.

All trials were started at 6:00 a.m., and DMDS and DMTS were collected immediately after the start of the experiment and 12 and 30 h later by static headspace sampling using disc‐shaped adsorbents, MonoTrap DCC18 (diameter, 1.0 cm; thickness, 0.1 cm; GL Sciences, Tokyo, Japan), which has a high surface area silica monolith structure containing activated carbon. Depending on the expected concentrations of DMDS and DMTS based on the preliminary experiment, one or three adsorbents were used for different combinations of conditions and sampling timings. During sampling, vial lids filled with moistened tissue paper were removed, and the mesh screens covering the containers were replaced with a dedicated lid (Figure 1c). As the MonoTrap DCC18 has a 0.1 cm hole in the center (Figure 1e), a round head nail (diameter, 0.09 cm; length, 2.5 cm; Figure 1f) was inserted in the hole using a tweezer. Then, the tip of the nail with adsorbents was inserted 1.0 cm into a rubber stopper (top diameter, 1.4 cm; bottom diameter, 1.0 cm; Figure 1d), and the rubber stopper was tightly fitted in the hole of the lid of each container (Figure 1c). Six hours after introduction, the adsorbents were recovered from the containers, lids were replaced with mesh screens, and vial lids with moistened tissue paper were placed again. The recovered adsorbents were placed in 5 mL glass vials with 600 μL of hexane, and then DMDS and DMTS were extracted for 5 min using an ultrasonic cleaner (US‐2R, As One, Osaka, Japan).

Solvent‐extracted samples were analyzed using the gas chromatograph‐mass spectrometer (GCMS‐QP2010, Shimadzu, Kyoto, Japan) equipped with an Rtx‐5MS column (length, 30 m; diameter, 0.25 mm; film thickness, 0.25 μm; Restek, Pennsylvania). DMDS and DMTS standard solutions diluted to 0.01–0.5 ppm with hexane were also analyzed to make calibration curves for quantification. Standard and solvent‐extracted sample solutions (1 or 2 μL) were injected in the splitless mode at 200°C using an auto‐injector (AOC‐20i, Shimadzu, Kyoto, Japan). High‐purity helium (99.999%) was used as the carrier gas at a constant linear velocity of 41.7 cm per second. For the standards, the column temperature was maintained at 40°C for 3 min after the injection, then increased to 150°C at a rate of 10°C per min, and then maintained at 150°C for 2 min. The column temperature was increased to 250°C for sample analysis and held at 250°C for 5 min to elute contaminants from the column. The mass spectrometer was operated in the electron impact ionization mode with an ionization voltage of 70 eV and an ionization source temperature of 230°C. The quantification ions of DMDS and DMTS were monitored using the selected ion monitoring mode for molecular ions at m/z 94 and 126, respectively.

The DMDS and DMTS concentrations in the samples were quantified based on the calibration curves obtained from standard solutions. When the peak areas of the samples exceeded the range of the calibration curves, the samples were diluted with hexane and analyzed again. Because the dilution ratio and the number of adsorbents used differed among the samples, the DMDS and DMTS concentrations in the samples were corrected as follows: Cc = Co * (3/*N*) * *D*, where Cc is the corrected concentration, Co is the original concentration based on the calibration curves, *N* is the number of adsorbents used (i.e., 1 or 3), and *D* is the dilution ratio. When the peak areas of the samples were below the range of the calibration curves, the concentrations of the samples were treated as “not detected” (n.d.). Instrument detection limits (IDLs) and quantification limits (IQLs) were calculated as: IDL/IQL = *F* * (*σ*/*S*), where *F* is 3.3 for IDL and 10 for IQL, *σ* is the standard deviation of peak areas of 0.01 ppm DMDS and DMTS standard solutions (based on 10 measurements), and *S* is the slope of the calibration curve.

### 2.4 Effects of DMDS and DMTS on the attraction of carrion insects

To examine whether carrion insects respond to the DMDS and DMTS, a field experiment using pitfall traps was conducted in the artificial forest of the northern part of Chiba Prefecture, eastern Japan (35°39′22″ N, 140°14′52″ E) from October 10 to October 15, 2013. A detailed description of this site has been made previously (Ito, 2020). Before the experiment, the 24 experimental locations used to place traps were determined linearly on the forest floor at intervals of 25 m. Traps consisting of a cylindrical plastic container (diameter, 12.9 cm; height, 5.7 cm) with a 4 cm hole in the center of the upper surface were used (Figure S1a). Two flexible rectangular plastic plates (length, approximately 3.0 cm; width, approximately 0.7 cm) were attached to the inside of the containers in a cross to cover the hole. Plastic plates were prepared to prevent the captured insects from escaping.

At 12:00 p.m., traps were buried at each experimental location such that the top was level with the ground and the upper surface was covered with soil and leaf litter (Figure S1b). Then, a 1.5‐mL microtube containing one of the following baits was placed in the traps: (i) 40 μL DMDS + 40 μL DMTS (DMDS + DMTS), (ii) 1 mL of hexane (hexane), and (iii) an empty microtube (control). Hexane was selected as the irrelevant odor to evaluate the response of carrion insects to the odor itself. To enable permanent evaporation during the capture period, a 2 mm hole was made for DMDS + DMTS and the control microtube, and a 0.5 mm hole was made in the hexane microtube. A paper plate fixed with wooden stakes was placed next to the traps to facilitate trap identification (Figure S1b). One to four hours after placement, we observed each trap for 5 min and recorded the presence or absence of adult flies attracted to the trap bodies and paper plates. After 12 h following placement, all traps were recovered, and the number of species and individuals captured by each trap was recorded. Insects were identified at species, genus, or family levels based on specimens and photographs, and feeding habitats on carcasses were presumed based on previous observations (Ito, 2020) and existing literature (Table S1). Traps were placed at all experimental locations such that the same bait types were not adjacent. The trial was repeated three times every other day, and each type of bait was placed once at each experimental location throughout the trials.

### 2.5 Statistical analyses

All statistical analyses were performed using R version 4.2.0 (R Core Team, 2022). To compare the DMDS and DMTS concentrations under different experimental conditions (i.e., rat carcass, damaged carcass, carcass with feeding, and *Ne. japonica*) and sampling times (i.e., 0, 12, and 30 h), the Tukey–Kramer test was performed using the glht function in the multcomp package (Hothorn et al., 2008) without evaluation using analysis of variance. In these analyses, pairwise comparisons were conducted among the condition and sampling time combinations that could quantify the concentration using the absolute calibration curve method in at least two of three trials.

The total number of species and individuals captured by each pitfall trap was analyzed using generalized linear mixed models (GLMMs) with the glmer function in the lme4 package (Bates et al., 2015). These analyses treated the total number of species and individuals as dependent variables, and a Poisson error distribution with a log‐link function was used. Bait type was treated as an independent variable after conversion into dummy variables. As a dummy variable for DMDS + DMTS, a dummy variable of 1 was assigned to traps using DMDS + DMTS, and a dummy variable of 0 was assigned to other traps. As a dummy variable for hexane, a dummy variable of 1 was assigned to traps using hexane, and a dummy variable of 0 was assigned to other traps. Each trial was treated as a random effect. The significance of the independent variables was evaluated by a type II ANOVA (type II Wald chi‐square tests) using the Anova function in the car package (Fox & Weisberg, 2019). We also analyzed capture rates for each taxon between different bait types using Fisher's exact test. The attraction rates of adult flies to different baits were compared using Fisher's exact test.

## 3 RESULTS

### 3.1 Effect of *Ne. japonica* on DMDS and DMTS emissions from carcasses

**FIGURE 2:**
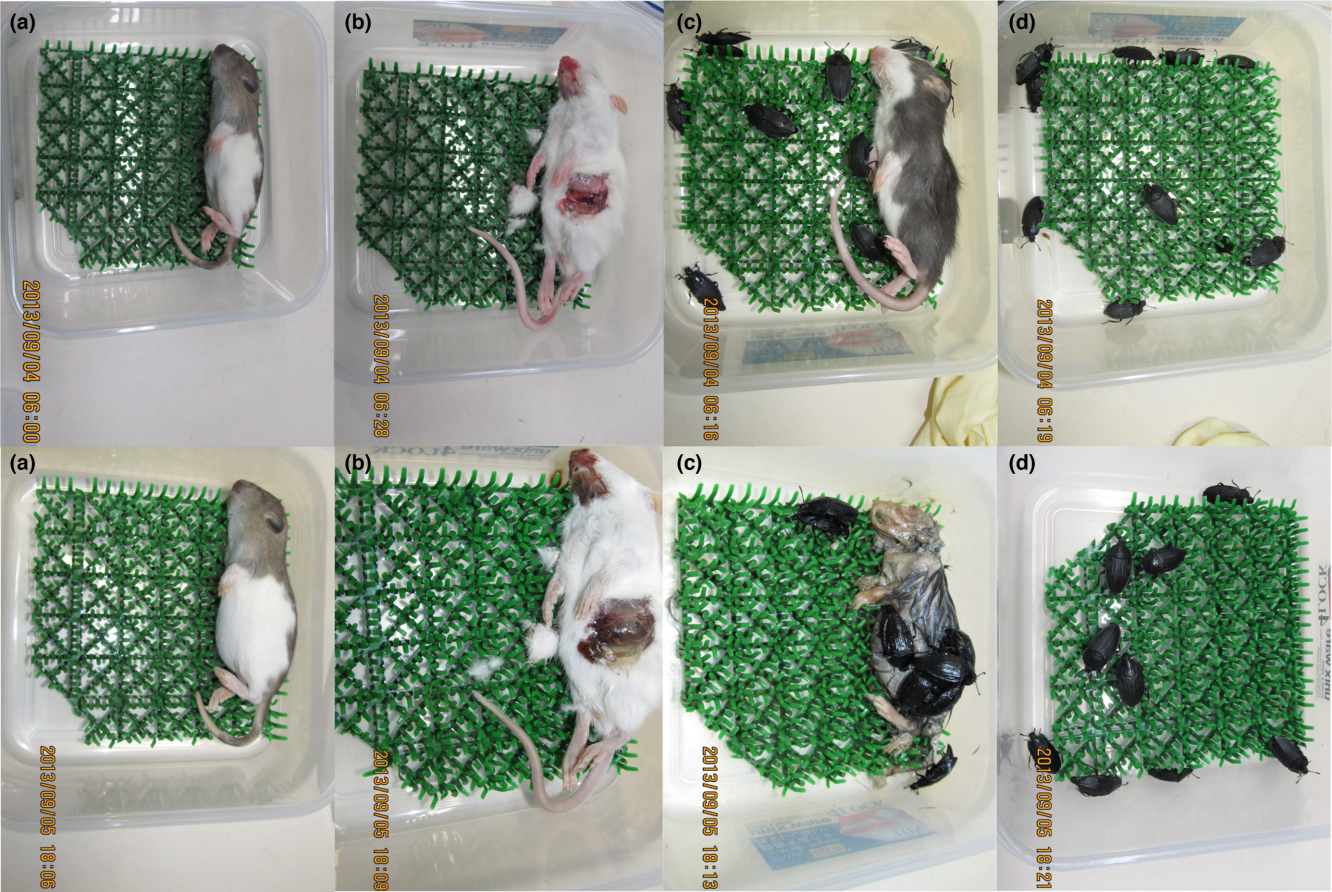
Changes of each condition (i.e., rat carcass (a), damaged carcass (b), carcass with feeding (c), and *Necrophila japonica* (d)) from the start (after 0 h, upper row) to the end (after 36 h, lower row) in the trial 1. The dates and times at which each picture was taken are shown at the bottom left.

After 36 h (i.e., at the end of the trials), the carcasses fed on by *Ne. japonica* presented a wet appearance, likely because of their feces, and the internal tissue was exposed (Figure 2c). However, carcasses that were not fed on by *Ne. japonica* exhibited slight bloating, whereas no other noticeable changes were observed (Figure 2a,b).

**FIGURE 3:**
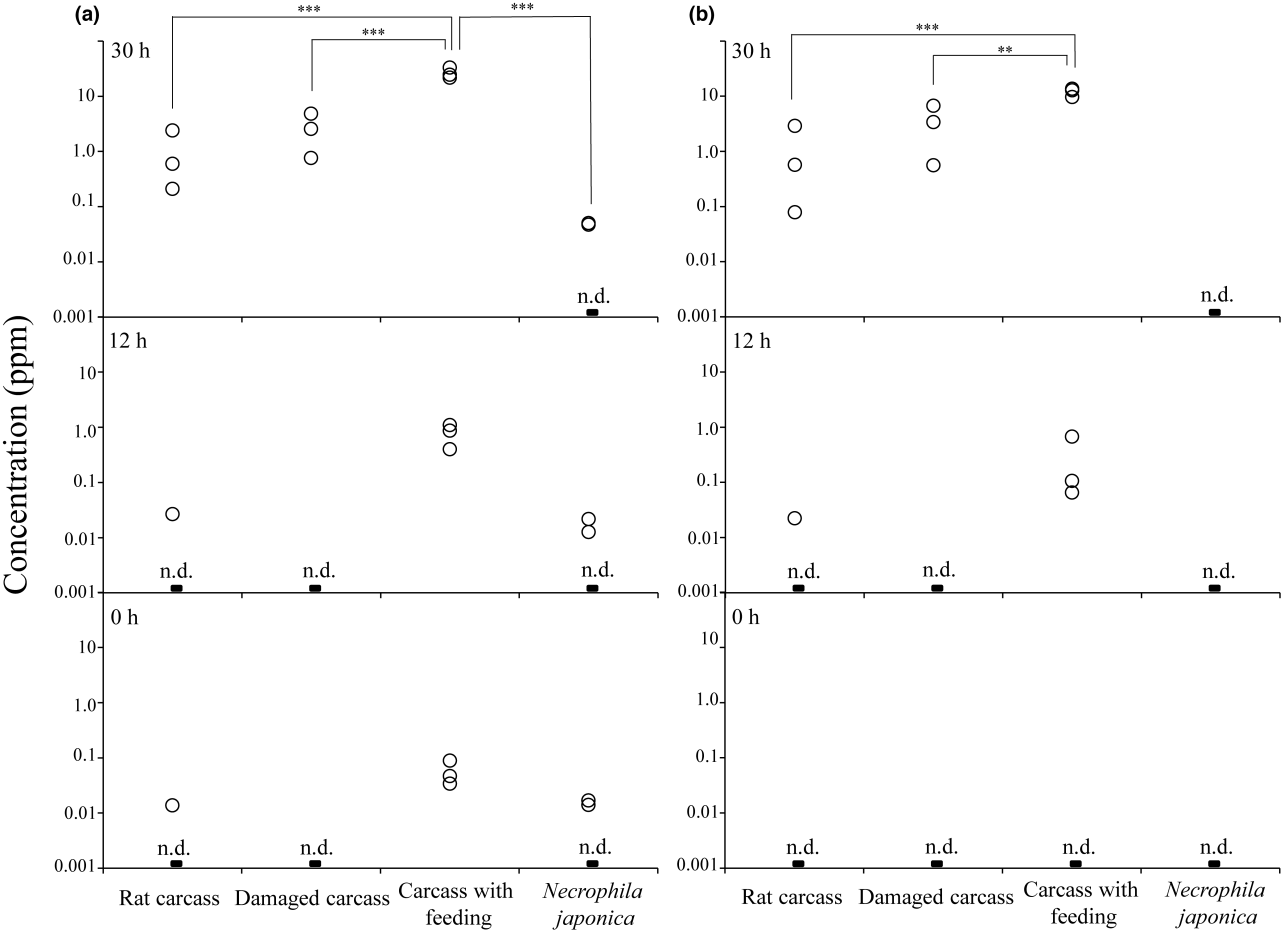
Concentrations (ppm) of dimethyl disulfide (a) and dimethyl trisulfide (b) under different conditions at different time points in the laboratory experiment. Each condition was tested three times. Differences among the different conditions were compared using Tukey–Kramer tests (***p < .001; **p < .01). n.d., not detected.

In the empty container, without a rat carcass or *Ne. japonica*, DMDS and DMTS peaks were not detected (Figure S2f). In contrast, DMDS and DMTS were detected under other conditions (Figures S2 and S3) and quantified using calibration curves (Figure S4). The IDLs and IQLs for DMDS were 0.0022 and 0.0067 ppm, respectively, and those for DMTS were 0.0014 and 0.0043 ppm, respectively. DMDS was quantified after 30 h in the carcasses that were not fed on by *Ne. japonica* except for one trial but was quantified immediately after the start of the trials in the carcasses fed on by *Ne. japonica* and increased over time (Figure 3a, Table S2). The DMDS concentration in carcasses with feeding was significantly higher after 30 h than at 0 and 12 h (Tukey–Kramer test, for all comparisons, *p* < .001). Moreover, after 30 h, carcasses fed on by *Ne. japonica* showed significantly higher DMDS concentrations than those of the same age under all other conditions (Tukey–Kramer test, for all comparisons, *p* < .001). There were no significant differences in DMDS concentrations between the 12‐h‐old carcasses with feeding and the 30‐h‐old rat carcasses or damaged carcasses (Tukey–Kramer test, for all comparisons, *p* > .05). Although there were no significant differences between the sampling times (Tukey–Kramer test, for all comparisons, *p* > .05), DMDS was also present, but approximately 100 times lower amount detected with *Ne. japonica* only than in other conditions after 30 h (Figure 3a, Table S2).

DMTS was quantified after 12 or 30 h only under conditions using rat carcasses (Figure 3b, Table S3). After 30 h, fed carcasses showed significantly higher DMTS concentrations than those of the same age under other conditions (Tukey–Kramer test: carcass with feeding vs. rat carcass, *t* = 6.604, *p* < .001; carcass with feeding vs. damaged carcass, *t* = 5.157, *p* = .0038). Moreover, the DMTS concentration in carcasses that were fed on was significantly higher after 30 h than after 12 h (Tukey–Kramer test, *t* = 7.151, *p* < .001). There were no significant differences in DMTS concentrations between 12‐h‐old fed carcasses and 30‐h‐old rat carcasses or damaged carcasses (Tukey–Kramer test, for all comparisons, *p* > .05). Unlike DMDS, DMTS was not quantified in the condition using only *Ne. japonica* (Figure 3b, Table S3). The results of all possible pairwise comparisons of DMDS and DMTS concentrations based on the Tukey–Kramer test are shown in Tables S4 and S5.

### 3.2 Effects of DMDS and DMTS on the attraction of carrion insects

**FIGURE 4:**
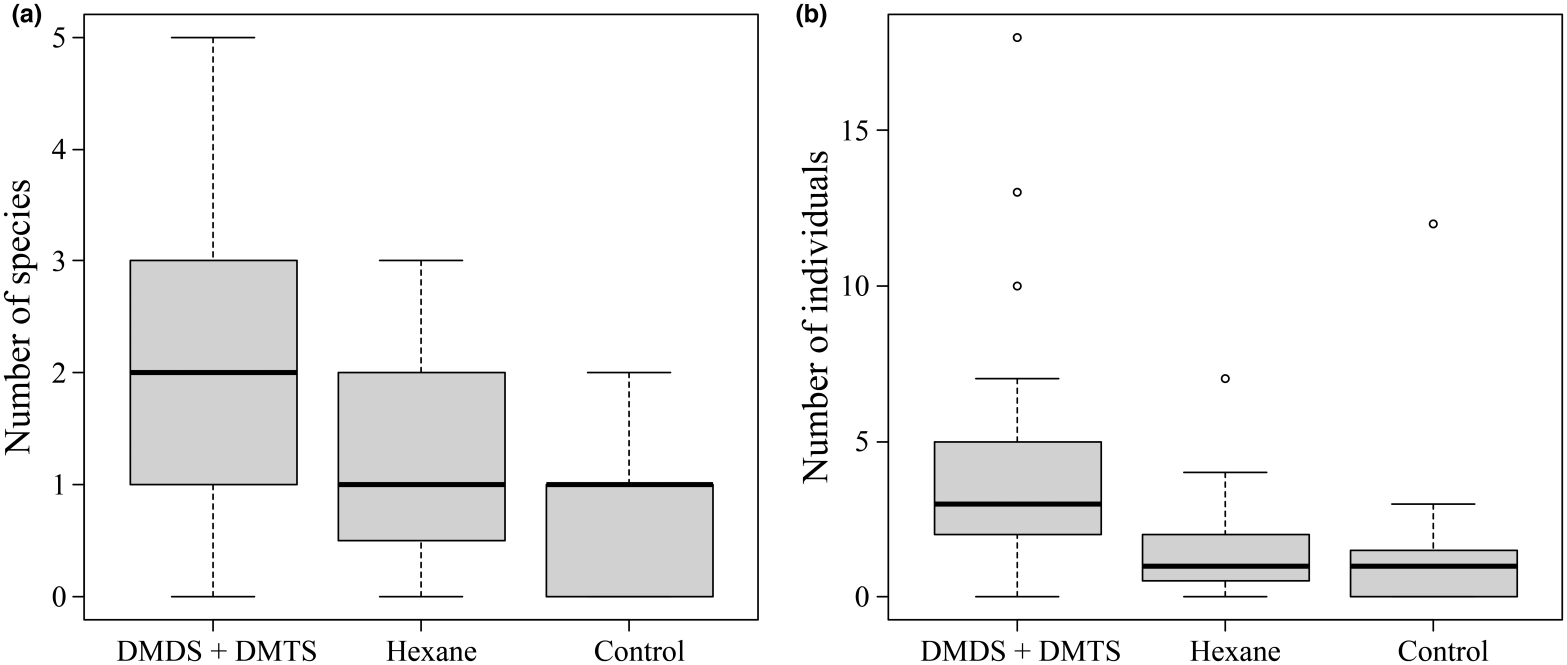
The total number of species (a) and total number of individuals (b) captured by each trap. dimethyl disulfide (DMDS) + dimethyl trisulfide (DMTS), traps baited with microtubes containing 40 μL DMDS and 40 μL DMTS; hexane, traps baited with microtubes containing 1 mL hexane; control, traps baited with empty microtubes.

Although no *Ni. concolor* individuals were captured, the field experiment using three types of bait captured 16 taxa belonging to 5 orders and 11 families (Table 1). DMDS and DMTS positively affected the total number of species (Figure 4a; GLMM and type II ANOVA, dummy variable for DMDS + DMTS, $\chi_{1}^{2}$ = 15.6228, *p* < .001) and the total number of individuals (Figure 4b; GLMM and type II ANOVA, dummy variable for DMDS + DMTS, $\chi_{1}^{2}$ = 32.689, *p* < .001) captured. In contrast, hexane did not affect the total number of species (Figure 4a; GLMM and type II ANOVA, dummy variable for hexane, $\chi_{1}^{2}$ = 1.9728, *p* = .16) and the total number of individuals (Figure 4b; GLMM and type II ANOVA, dummy variable for hexane, $\chi_{1}^{2}$ = 0.486, *p* = .49) captured.

**TABLE 1:** Number of traps that attracted each taxon.

Order	Family	Taxon	DMDS + DMTS	Hexane	Control	*p*‐Value	Presumed feeding habitat
Dermaptera	Anisolabididae	*Anisolabella marginalis*	2			.32	Necrophagous/Predatory
Orthoptera	Rhaphidophoridae	*Diestrammena* sp.	8			<.001	Necrophagous
	Gryllidae	Gryllidae sp.	4	1		.12	
Diptera	Calliphoridae and/or Sarcophagidae	Adult fly	23			<.001	Necrophagous
		Fly larvae	2			.32	Necrophagous
Coleoptera	Carabidae	*Brachinus scotomedes*	1			1	
		*Haplochlaenius costiger*	1		1	1	Predatory
		*Carabus* spp.	5	6	3	.65	Predatory
		*Synuchus* spp.	12	11	3	.011	Predatory
	Leiodidae	Cholevinae sp.	3			.10	Necrophagous
	Staphylinidae	*Necrophila japonica*	2	1		.77	Necrophagous/Predatory
	Geotrupidae	*Phelotrupes laevistriatus*	1			1	Necrophagous
	Scolytidae	Scolytidae sp.	1			1	
Hymenoptera	Formicidae	*Brachyponera chinensis*	3	3	1	.69	Predatory
		*Paratrechina flavipes*	7	6	8	.95	Necrophagous/Predatory
		*Pheidole fervida*	2	1	4	.49	Necrophagous/Predatory

*Note*: DMDS + DMTS, traps baited with 40 μL DMDS and 40 μL DMTS; hexane, traps baited with 1 mL hexane; Control, traps baited with an empty microtube. A total of 24 traps per bait type were used. *p*‐Values are based on Fisher's exact tests that compared attraction rates between different bait types.

Eight taxa were attracted only to traps baited with DMDS and DMTS, and of these, the attraction rates of *Diestrammena* sp. (Rhaphidophoridae) and adult flies (Calliphoridae and/or Sarcophagidae) to traps baited with DMDS and DMTS were significantly higher (Table 1; Fisher's exact tests, for all comparisons, *p* < .001). Attraction rates of *Synuchus* spp. (Carabidae) were significantly higher in the traps baited with DMDS + DMTS or hexane (Table 1; Fisher's exact test, *p* = .011). No taxa were attracted only to hexane or the empty microtubes (control).

## 4 DISCUSSION

Our results showed that carcasses fed on by *Ne. japonica*, an early colonist, accelerated the emissions of DMDS and DMTS and that DMDS and DMTS can contribute to attracting carrion insects. In the laboratory experiment, although the concentrations of DMDS and DMTS emitted from the rat carcasses increased over time, the carcasses fed on by *Ne. japonica* had higher concentrations of DMDS and DMTS than normal or damaged carcasses that had passed the same amount of time (Figure 3). In the field experiment, traps baited with DMDS and DMTS attracted more necrophagous insect species and individuals than traps that were not baited (Table 1, Figure 4). However, the results of the laboratory experiment were based on small sample sizes (i.e., three replications per condition). Moreover, the field experiment did not directly evaluate the effect of carcass modification by *Ne. japonica* on community assembly, as we did not measure nor relate the emission rates of DMDS and DMTS in the two experiments. Therefore, further verification of the reproducibility of our results and the relevance of laboratory and field experiments is needed. However, the evidence obtained from our experiments suggests that modifications by early colonists that accelerated the emissions of DMDS and DMTS may promote community assembly during carrion insect succession.

The result of the laboratory experiment may well explain our previous finding (Ito, 2022) that *Ne. japonica* promotes the discovery of carcasses by *Ni. concolor*. However, *Nicrophorus* beetles were not attracted to traps baited with DMDS and DMTS (Table 1). A recent study showed that the attraction of *Ni*. *orbicollis* in search of small fresh carcasses for reproduction is promoted by DMDS but deterred by DMTS, which is abundantly released in the later stages of decomposition (Trumbo & Steiger, 2020). von Hoermann et al. (2016) suggested that *Ni*. *vespilloides* females with immature ovaries search large carcasses in the later stages of decomposition for feeding using DMTS. Although maturity was not recorded, Podskalská et al. (2009) captured *Ni*. *vespillo* using traps baited with DMDS and DMTS and suggested that they have a synergistic effect. Therefore, the response of *Nicrophorus* beetles may differ depending on the combination of compounds (i.e., DMDS or DMTS), species, and maturity. Moreover, the final entry of *Nicrophorus* beetles attracted by DMDS and DMTS into the traps may require a more complex blend of carcass odors (Trumbo & Dicapua, 2021). The month of October, when the field experiment was conducted, was the breeding season for *Ni*. *quadripunctatus*, which breeds predominantly from April to May and September to October, but it may not have been for *Ni. concolor*, which breeds predominantly from July to September, with a peak in August (Nagano & Suzuki, 2003b). Therefore, to evaluate the effect of DMDS and DMTS on the attraction of *Nicrophorus* beetles, further experiments that consider the breeding season and use actual carcasses supplemented with DMDS and DMTS individually, as in Trumbo and Dicapua (2021), are needed.

Although we focused on DMDS and DMTS as important volatile attractants for major carrion insects (Verheggen et al., 2017), *Nicrophorus* beetles respond to volatile organic compounds other than DMDS and DMTS (Kalinová et al., 2009; von Hoermann et al., 2016). Recently, methyl thiocyanate has been identified as an important attractant (Trumbo & Steiger, 2020). Moreover, the synergy, emission rates (volatility), and relative concentrations of multiple compounds are important as well (Podskalská et al., 2009; Trumbo & Dicapua, 2021; Trumbo & Newton, 2022). Therefore, to elucidate the comprehensive mechanisms that attract carrion insects to carcasses, dose–response tests and experiments considering relative concentrations of various volatile organic compounds are also needed.

To understand the mechanisms of carrion insect succession, demonstration of the effects of pioneer species on later colonists (interspecific interaction) and the carcasses (modification) is required (Michaud & Moreau, 2017). However, empirical research on interspecific interactions and modification of volatile organic compounds have been conducted independently or outside the context of carrion insect succession. Several experimental studies that temporarily prevented colonization of carcasses by early colonists showed that successional trajectories were changed by these treatments and suggested the existence of some interspecific interaction between early and later colonists (Kadlec et al., 2019; Pechal, Benbow, et al., 2014). Moreover, experimental studies that manipulated the utilization of carcasses by early colonists or specific species showed that their carcass utilization hastens the colonization by later or other species (Ito, 2022; Michaud & Moreau, 2017; Trumbo & Sikes, 2021) and demonstrated positive interspecific interactions that may be consistent with the processing chain (Heard, 1994) or facilitation model (Connell & Slatyer, 1977). However, studies focusing on the modification of volatile organic compounds have revealed that carcass utilization by early colonists or specific species affects the emissions of volatile organic compounds from carcasses (Martin et al., 2019; Recinos‐Aguilar et al., 2019; Trumbo et al., 2021). Our present and past results (Ito, 2022) support the results of the previous studies outlined above and showed that interspecific interactions and modification of volatile organic compounds are driven by an early colonist, *Ne. japonica*.

Because DMDS and DMTS are emitted from carcasses due to the degradation of sulfur‐containing amino acids by microorganisms (Kadota & Ishida, 1972; Paczkowski & Schütz, 2011), ultimately, *Ne. japonica* is thought to have affected microbial communities. Previous studies have suggested that artificial physical damage enhances microbial colonization of carcasses (Brodie et al., 2016; Trumbo, 2017) and that physical damage by insect feeding accelerates the emissions of DMDS and DMTS (Chen et al., 2020; Recinos‐Aguilar et al., 2019). Soil is regarded as the main source of the microbial communities formed on vertebrates after death (Metcalf et al., 2016). Therefore, physical damage is considered to play an important role in the colonization of carcasses by soil microorganisms. In the present study, although DMDS and DMTS emissions showed increasing trends in the damaged carcasses compared to those in the normal rat carcasses after 30 h, the differences were not statistically significant (Figure 3, Tables S2 and S3). Because our experiment was performed without soil in a relatively dry and clean laboratory environment, we may have underestimated the effect of physical damage on microbial colonization and activity. Moreover, the lack of significance in damaged carcasses may be related to the lack of statistical power resulting from the small sample size of three replicates per condition. However, the fact that the carcasses fed on by *Ne. japonica* did increase emissions of DMDS and DMTS despite the absence of soil shows that *Ne. japonica*, as well as the soil, can be sources of microorganisms.

Insects are known to directly affect microbial communities in carcasses. Adult flies have microorganisms on their proboscises, legs, feces, and eggs (Barro et al., 2006; Brundage et al., 2017) and introduce them into carcasses that they colonize (Metcalf et al., 2016). Adult *Nicrophorus* beetles, which process a small carcass into a round shape and raise offspring on it (Scott, 1998), regulate microbial communities on the carcass to conditions suitable for the offspring by applying oral and anal secretions containing antimicrobial compounds (Degenkolb et al., 2011; Hoback et al., 2004) and symbiotic gut microorganisms (Miller et al., 2019; Vogel et al., 2017). *Necrophila* beetles, including *Ne. japonica*, have symbiotic gut microorganisms that are partly shared with *Nicrophorus* beetles, such as Clostridiaceae and Xanthomonadaceae (Kaltenpoth & Steiger, 2014; Kudo et al., 2019), and several species belonging to these families can produce DMDS (Cernosek et al., 2020; Segal & Starkey, 1969). Therefore, *Ne. japonica* may have accelerated the emissions of DMDS and DMTS from carcasses by physical modification, as well as by introducing symbiotic gut microorganisms into the feeding site through contact and feces (Figure 2). Moreover, the weak emission of DMDS under conditions using only *Ne. japonica* may have originated from their feces, a product of the degradation of sulfur‐containing amino acids by symbiotic microorganisms in the digestive tract. To link the present results to microorganisms, it is necessary to isolate microorganisms from carcasses and the digestive tract of *Ne. japonica* and evaluate their DMDS and DMTS emissions.

Traps baited with DMDS and DMTS significantly or specifically attracted taxa that were presumed to be necrophagous (Table 1). Species related to these taxa (Rhaphidophoridae, Calliphoridae, Leiodidae, and Geotrupidae) are known to be attracted to carcasses (Ito, 2020; Kadlec et al., 2019; Kočárek, 2003; Matuszewski et al., 2008) or sulfur‐containing volatile organic compounds (Frederickx et al., 2012; Navarro‐Llopis et al., 2021; Stensmyr et al., 2002; Weithmann et al., 2020). Although carrion insects have been regarded as important drivers of decomposition (Parmenter & MacMahon, 2009; Payne, 1965; Simmons et al., 2010), Barton and Evans (2017) revealed the importance of species composition and the negative effects of predatory species on decomposition rates. *Necrophila japonica* feed on fly larvae as well as vertebrate carcasses (Watahiki & Sasakawa, 2019). Therefore, *Ne. japonica* may have a positive effect on community assembly and decomposition rate through the attraction of necrophagous insects and carcass feeding, while also negatively affecting them through predation on fly larvae.

A recent study revealed the existence of interdependence among bacteria, volatile organic compounds, and flies, as well as temporal and spatial abiotic factors that affect the strength of this interdependence (von Hoermann et al., 2022). Although we did not directly evaluate bacteria, present and previous studies (Ito, 2022) have shown that *Ne. japonica*, an early colonist, may affect volatile organic compounds produced by microorganisms and other carrion insects. Therefore, future forensic and carrion‐focused ecological studies should consider the existence of species that influence the strength of this interdependence.

## 5 CONCLUSION

Our results suggest that the pioneer carrion beetle, *Ne. japonica*, may promote community assembly during carrion insect succession by accelerating the emissions of DMDS and DMTS, which attract necrophagous insects; however, further verification of the reproducibility of the results and consideration of actual DMDS and DMTS emission rates are needed. Although further experiments on the response of *Nicrophorus* beetles to DMDS and DMTS are needed, by combining the present results with previous findings (Ito, 2022), which demonstrated that *Ne. japonica* promotes the attraction of a later colonist, *Ni. concolor*, to carcasses, we also showed the effects of pioneer species on carrion insect succession from the aspects of interspecies interactions and carcass chemical modification. Because DMDS and DMTS are emitted from carcasses because of microbial activity, *Ne. japonica* is thought to have accelerated the DMDS and DMTS emissions by introducing microorganisms into the feeding site through contact and feces. Bacteria, volatile organic compounds, and flies are interdependent in carrion ecosystems (von Hoermann et al., 2022). Our present and previous studies (Ito, 2022) show that *Ne. japonica* may affect each component of this interdependence and emphasize the importance of considering such species in future forensic and carrion‐focused ecological studies.

## AUTHOR CONTRIBUTIONS

**Minobu Ito:** Conceptualization (equal); formal analysis (equal); investigation (equal); methodology (equal); project administration (lead); resources (equal); visualization (equal); writing – original draft (lead); writing – review and editing (equal). **Atsuko Nishigaki:** Conceptualization (supporting); formal analysis (equal); investigation (equal); methodology (equal); resources (equal); visualization (equal); writing – original draft (supporting); writing – review and editing (equal). **Masami Hasegawa:** Conceptualization (equal); methodology (equal); visualization (supporting); writing – review and editing (equal).

## CONFLICT OF INTEREST STATEMENT

The authors have no relevant financial or nonfinancial interests to disclose.

## Supporting information


Appendix S1

## Data Availability

The data that support the findings of this study are openly available in figshare (10.6084/m9.figshare.23674620).
