# Supplementary material for: The effect of pioneer carrion beetles on the emission of volatile organic compounds and carrion insect community assembly
Source: Ecol Evol. 2023 Dec 12;13(12):e10818. doi: 10.1002/ece3.10818 (PMC10714124; doi:10.1002/ece3.10818)
Supplement: Supplementary file 1 — Appendix S1 [file ECE3-13-e10818-s001.docx]

**Supplementary Information for the *Ecology and Evolution***

**The effect of pioneer carrion beetles on the emission of volatile organic compounds and carrion insect community assembly**

MINOBU ITO*, ATSUKO NISHIGAKI and MASAMI HASEGAWA

**Department of Biology, Graduate School of Science, Toho University, 2-2-1 Miyama, Funabashi, Chiba 274-8510, Japan*

E-mail: itominobusati@gmail.com

ORCID: 0000-0003-2451-624X

**Supplementary Figures**

**
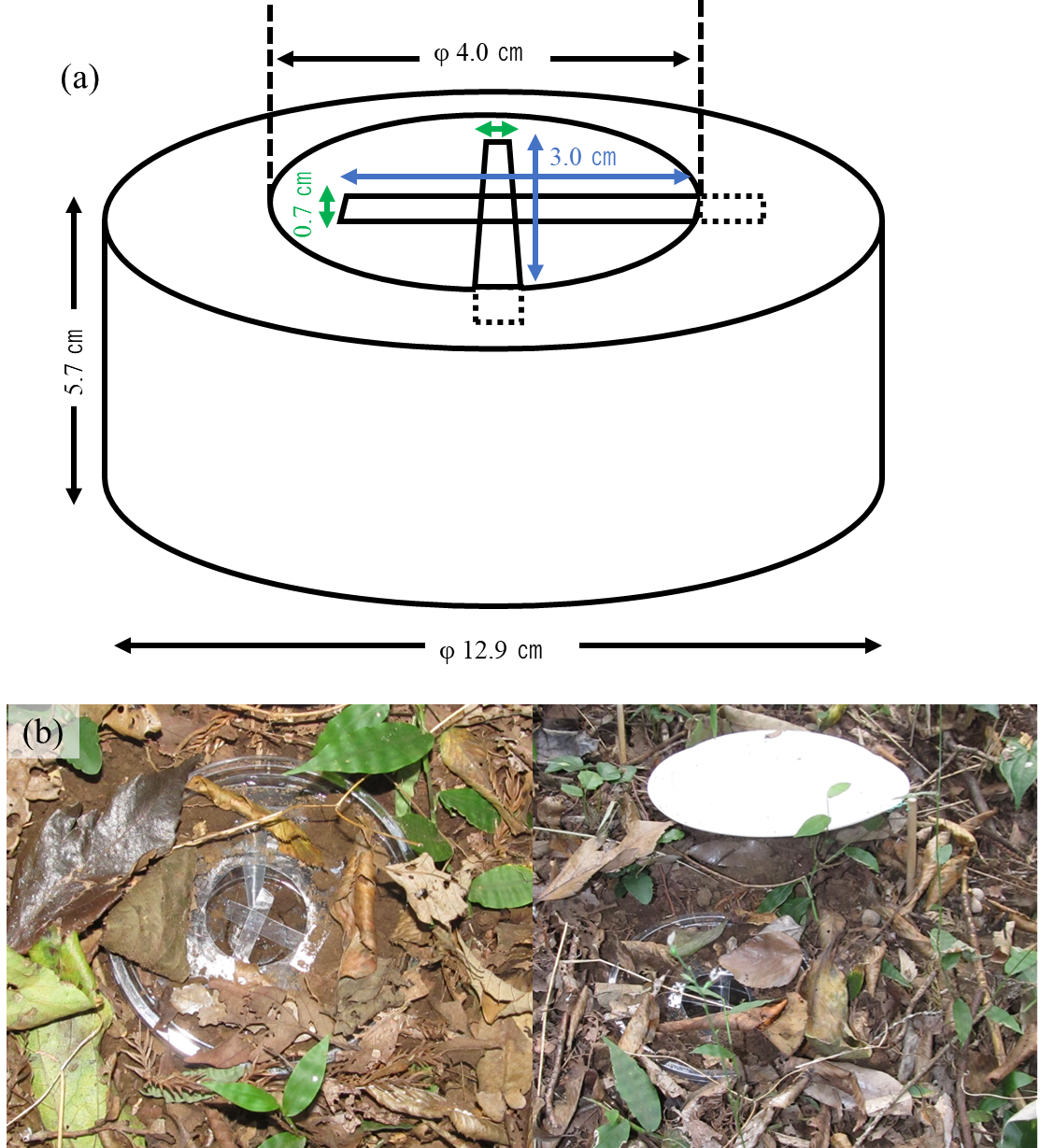
**

**Figure S1** Schematic diagram (a) and pictures (b) of pitfall traps used to examine the responses of carrion insects to dimethyl disulfide (DMDS) and dimethyl trisulfide (DMTS). One of three types of microtube (i.e., microtube containing 40 μL of DMDS and 40 μL of DMTS, microtube containing 1 mL of hexane, or empty microtube) was placed inside the traps. Microtubes were placed approximately 7.7 cm below the soil surface (i.e., trap height, 5.7 cm + soil and leaf litter that cover the trap surface, approximately 2.0 cm).


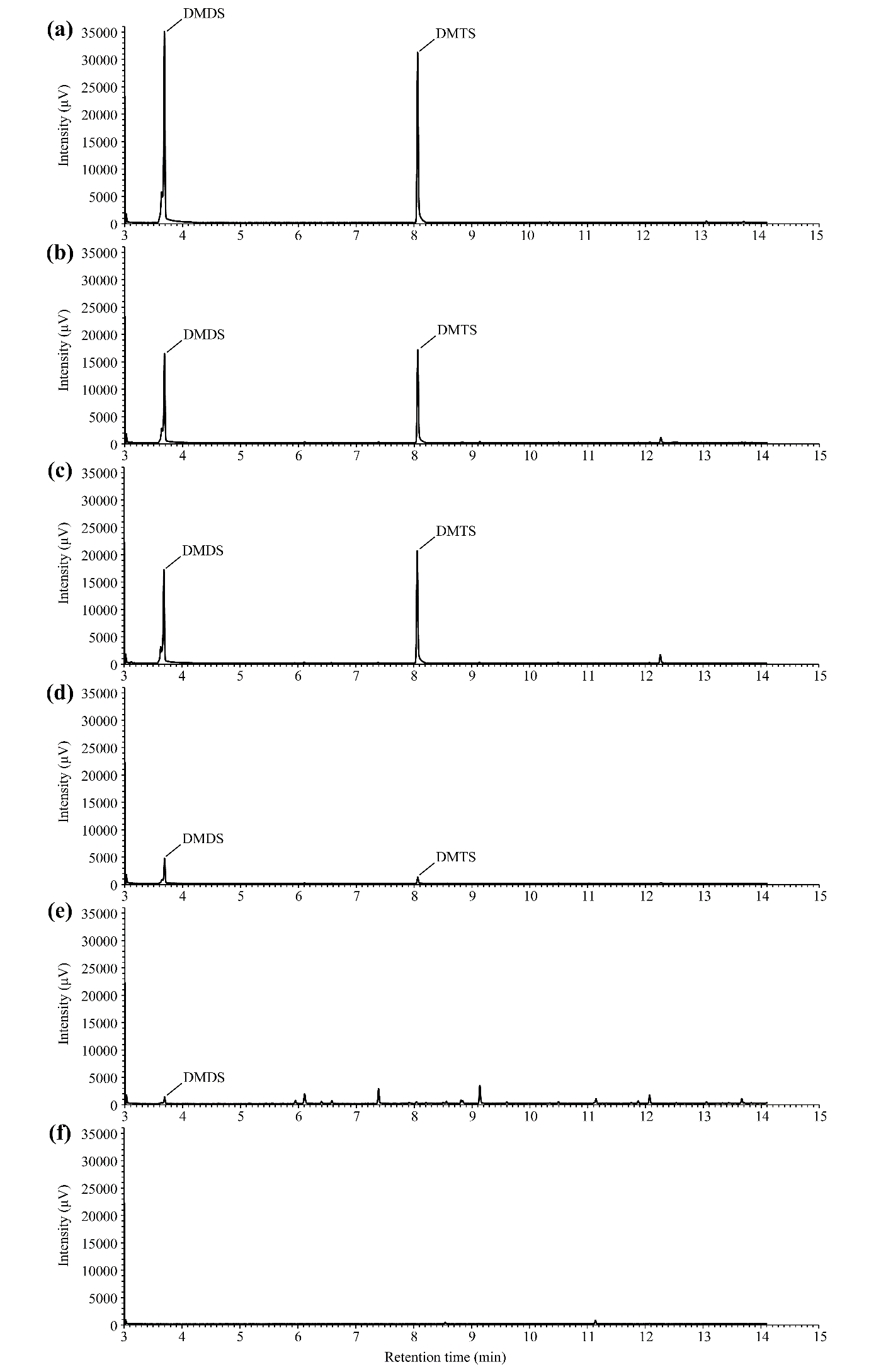


**Figure S2** Typical chromatograms of dimethyl disulfide (DMDS) and dimethyl trisulfide (DMTS) obtained from the 0.5 ppm standard solution (a), 30-hour-old samples of rat carcass (b), damaged carcass (c), carcass with feeding (d), *Necrophila japonica* (e) and an empty container (f). The fed carcass samples (d) diluted 100 times with hexane.

**
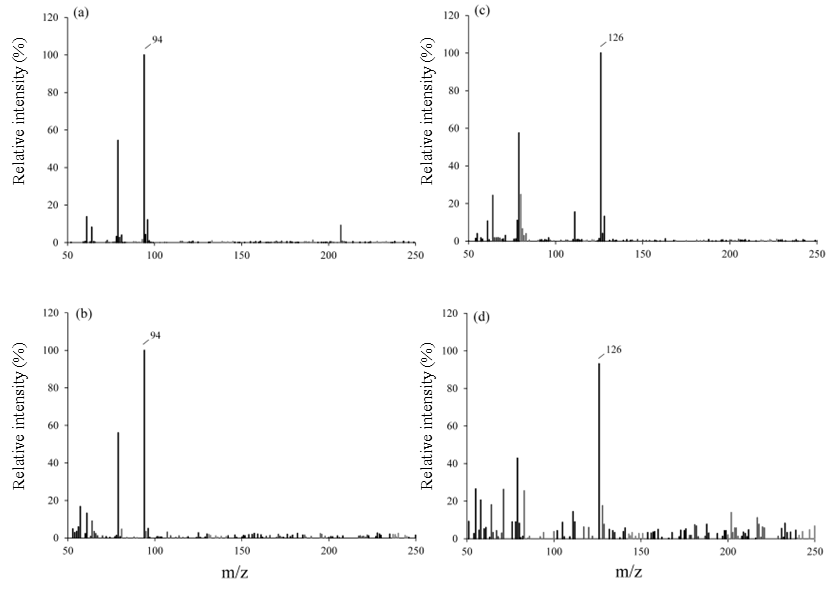
**

**Figure S3** Mass spectra of dimethyl disulfide obtained from 0.3 ppm of standard solution (a) and 30-hour-old rat carcass (b), and mass spectra of dimethyl trisulfide obtained from 0.3 ppm of standard solution (c) and 30-hour-old rat carcass (d).

**
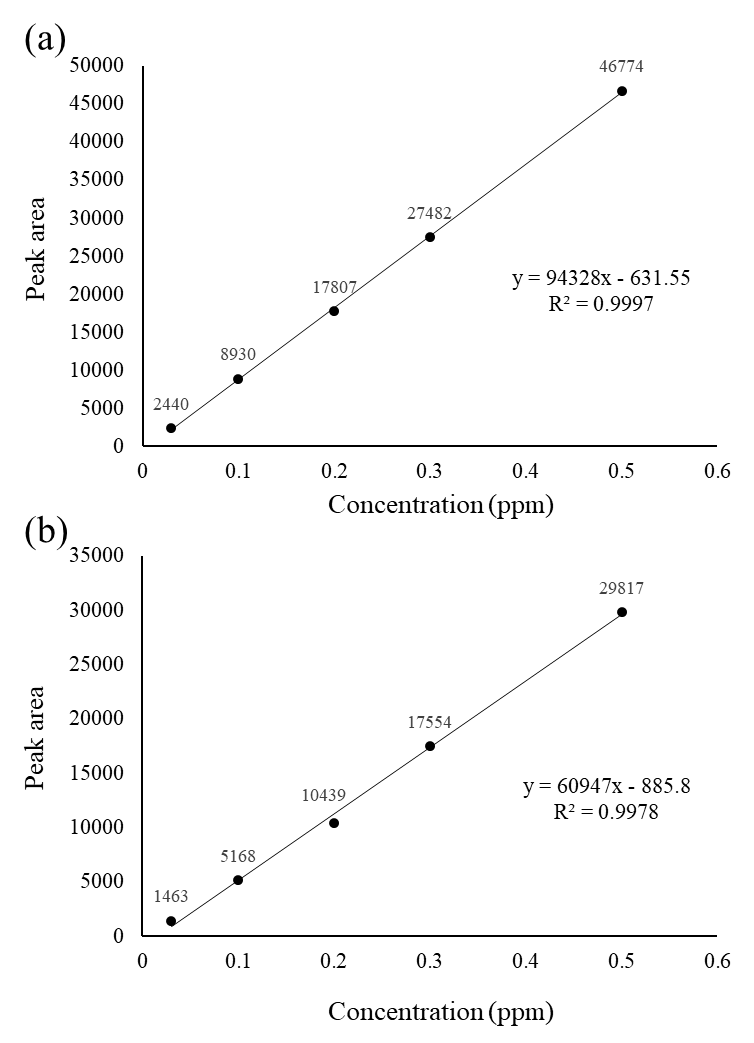
**

**Figure S4** Calibration curves used for quantification of dimethyl disulfide (a) and dimethyl trisulfide (b) in 30-hour-old samples in Trial 2.

**Supplementary Tables**

**Table S1** References used to presume the feeding habitats of insects captured by traps.

| Order | Family | Taxon | Presumed feeding habitats | Reference |
| --- | --- | --- | --- | --- |
| Dermaptera | Anisolabididae | *Anisolabella marginalis* | Necrophogous / Predatory | Nishikawa M (2006) Life of earwigs. The nature and insects 41(9):4–8 (in Japanese)  Nishikawa M (2016) Dermaptera. In: Orthopterological Society of Japan (ed) The standard of Polyneoptera in Japan. Gakken, Tokyo, pp 170–186 (in Japanese) |
| Orthoptera | Rhaphidophoridae | *Diestrammena* sp. | Necrophogous | Kano Y, Kawai M, Ichikawa A, Tominaga O, Murai T (2016) Orthoptera. In: Orthopterological Society of Japan (ed) The standard of Polyneoptera in Japan. Gakken, Tokyo, pp 242–371 (in Japanese) |
|  | Gryllidae | Gryllidae sp. |  |  |
| Diptera | Calliphoridae and/or Sarcophagidae | Adult fly | Necrophogous | Ito M (2020) Study of community assembly patterns and interspecific interactions involved in insect succession on rat carcasses. Entomological Science 23(1):105–116 |
|  |  | Fly larvae | Necrophogous | Ito M (2020) Study of community assembly patterns and interspecific interactions involved in insect succession on rat carcasses. Entomological Science 23(1):105–116 |
| Coleoptera | Carabidae | *Brachinus scotomedes* |  |  |
|  |  | *Haplochlaenius costiger* | Predatory | Okuzaki Y, Tayasu I, Okuda N, Sota T (2010) Stable isotope analysis indicates trophic differences among forest floor carabids in Japan. Entomologia Experimentalis et Applicata 135(3):263–270 |
|  |  | *Carabus* spp. | Predatory | Okuzaki Y, Tayasu I, Okuda N, Sota T (2010) Stable isotope analysis indicates trophic differences among forest floor carabids in Japan. Entomologia Experimentalis et Applicata 135(3):263–270 |
|  |  | *Synuchus* spp. | Predatory | Okuzaki Y, Tayasu I, Okuda N, Sota T (2010) Stable isotope analysis indicates trophic differences among forest floor carabids in Japan. Entomologia Experimentalis et Applicata 135(3):263–270 |
|  | Leiodidae | Cholevinae sp. | Necrophogous | Hayashi N, Morimoto K (1986) Humans and beetles. In: Morimoto K, Hayashi N (ed) The coleoptera of Japan in color Vol. Ⅰ. Hoikusha Publishing, Osaka, pp 234–254 (in Japanese) |
|  | Staphylinidae | *Necrophila japonica* | Necrophogous  / Predatory | Ito M (2020) Study of community assembly patterns and interspecific interactions involved in insect succession on rat carcasses. Entomological Science 23(1):105–116  Watahiki Y, Sasakawa K (2019) Effects of diet on female fecundity and larval development in the carrion beetle *Necrophila japonica*. Entomologia Experimentalis et Applicata 167(2):85–90 |
|  | Geotrupidae | *Phelotrupes laevistriatus* | Necrophogous | Akamine M, Sato H (2011) Nich Segregation among three dung beetles (Scarabaeidae). Japanese journal of entomology 14(4):290–296 (in Japanese with English abstract) |
|  | Scolytidae | Scolytidae sp. |  |  |
| Hymenoptera | Formicidae | *Brachyponera chinensis* | Predatory | Terayama M, Kubota S, Eguchi K (2014) Encyclopedia of Japanese ants. Asakura Publishing, Tokyo, pp 278 (in Japanese) |
|  |  | *Paratrechina flavipes* | Necrophogous / Predatory | Terayama M, Kubota S, Eguchi K (2014) Encyclopedia of Japanese ants. Asakura Publishing, Tokyo, pp 278 (in Japanese)  Eubanks MD, Lin C, Tarone AM (2019) The role of ants in vertebrate carrion decomposition. Food Webs 18:e00109. |
|  |  | *Pheidole fervida* | Necrophogous / Predatory | Terayama M, Kubota S, Eguchi K (2014) Encyclopedia of Japanese ants. Asakura Publishing, Tokyo, pp 278 (in Japanese)  Eubanks MD, Lin C, Tarone AM (2019) The role of ants in vertebrate carrion decomposition. Food Webs 18:e00109. |

**Table S2** Concentration (PPM) of dimethyl disulfide under different conditions and sampling times (h).

|  |  | Sampling times (hours) | | |
| --- | --- | --- | --- | --- |
| Condition | Trial | 0 | 12 | 30 |
| Rat carcass | 1 | n.d. | n.d. | 0.21 |
|  | 2 | 0.014 | 0.027 | 2.4 |
|  | 3 | n.d. | n.d. | 0.60 |
| Damaged carcass | 1 | n.d. | n.d. | 0.77 |
|  | 2 | n.d. | n.d. | 2.6 |
|  | 3 | n.d. | n.d. | 4.9 |
| Carcass with feeding | 1 | 0.089 | 0.40 | 33 |
|  | 2 | 0.034 | 1.1 | 22 |
|  | 3 | 0.047 | 0.87 | 25 |
| *Necrophila japonica* | 1 | 0.014 | n.d. | 0.051 |
|  | 2 | n.d. | 0.013 | n.d. |
|  | 3 | 0.017 | 0.022 | 0.047 |

n.d., not detected.

**Table S3** Concentration (PPM) of dimethyl trisulfide under different conditions and sampling times (h).

|  |  | Sampling times (hours) | | |
| --- | --- | --- | --- | --- |
| Condition | Trial | 0 | 12 | 30 |
| Rat carcass | 1 | n.d. | n.d. | 0.079 |
|  | 2 | n.d. | 0.022 | 2.9 |
|  | 3 | n.d. | n.d. | 0.57 |
| Damaged carcass | 1 | n.d. | n.d. | 0.56 |
|  | 2 | n.d. | n.d. | 3.4 |
|  | 3 | n.d. | n.d. | 6.7 |
| Carcass with feeding | 1 | n.d. | 0.065 | 13 |
|  | 2 | n.d. | 0.67 | 9.7 |
|  | 3 | n.d. | 0.11 | 14 |
| *Necrophila japonica* | 1 | n.d. | n.d. | n.d. |
|  | 2 | n.d. | n.d. | n.d. |
|  | 3 | n.d. | n.d. | n.d. |

n.d., not detected.

**Table S4** Results of all possible pairwise comparisons of the concentration of dimethyl disulfide (DMDS) based on the Tukey–Kramer test.

| Condition 1 |  | Condition 2 | SE | t-value | P-value |
| --- | --- | --- | --- | --- | --- |
| 0 h carcass with feeding | vs | 0 h *Necrophila japonica* | 2.281344 | 0.018 | 1 |
| 0 h carcass with feeding | vs | 12 h carcass with feeding | 2.040496 | -0.360 | 1 |
| 0 h carcass with feeding | vs | 12 h *Necrophila japonica* | 2.281344 | 0.017 | 1 |
| 0 h carcass with feeding | vs | 30 h rat carcass | 2.040496 | -0.500 | 0.999 |
| 0 h carcass with feeding | vs | 30 h damaged carcass | 2.040496 | -1.317 | 0.876 |
| 0 h carcass with feeding | vs | 30 h carcass with feeding | 2.040496 | -12.987 | <0.001 |
| 0 h carcass with feeding | vs | 30 h *Necrophila japonica* | 2.281344 | 0.003 | 1 |
| 0 h *Necrophila japonica* | vs | 12 h carcass with feeding | 2.281344 | -0.340 | 1 |
| 0 h *Necrophila japonica* | vs | 12 h *Necrophila japonica* | 2.499087 | -0.001 | 1 |
| 0 h *Necrophila japonica* | vs | 30 h rat carcass | 2.281344 | -0.466 | 1 |
| 0 h *Necrophila japonica* | vs | 30 h damaged carcass | 2.281344 | -1.196 | 0.919 |
| 0 h *Necrophila japonica* | vs | 30 h carcass with feeding | 2.281344 | -11.634 | <0.001 |
| 0 h *Necrophila japonica* | vs | 30 h *Necrophila japonica* | 2.499087 | -0.013 | 1 |
| 12 h carcass with feeding | vs | 12 h *Necrophila japonica* | 2.281344 | 0.339 | 1 |
| 12 h carcass with feeding | vs | 30 h rat carcass | 2.040496 | 0.141 | 1 |
| 12 h carcass with feeding | vs | 30 h damaged carcass | 2.040496 | 0.957 | 0.973 |
| 12 h carcass with feeding | vs | 30 h carcass with feeding | 2.040496 | 12.627 | <0.001 |
| 12 h carcass with feeding | vs | 30 h *Necrophila japonica* | 2.281344 | -0.325 | 1 |
| 12 h *Necrophila japonica* | vs | 30 h rat carcass | 2.281344 | 0.465 | 1 |
| 12 h *Necrophila japonica* | vs | 30 h damaged carcass | 2.281344 | 1.195 | 0.919 |
| 12 h *Necrophila japonica* | vs | 30 h carcass with feeding | 2.281344 | 11.633 | <0.001 |
| 12 h *Necrophila japonica* | vs | 30 h *Necrophila japonica* | 2.499087 | 0.013 | 1 |
| 30 h rat carcass | vs | 30 h damaged carcass | 2.040496 | -0.817 | 0.989 |
| 30 h rat carcass | vs | 30 h carcass with feeding | 2.040496 | 12.487 | <0.001 |
| 30 h rat carcass | vs | 30 h *Necrophila japonica* | 2.281344 | 0.451 | 1 |
| 30 h damaged carcass | vs | 30 h carcass with feeding | 2.040496 | 11.670 | <0.001 |
| 30 h damaged carcass | vs | 30 h *Necrophila japonica* | 2.281344 | -1.181 | 0.923 |
| 30 h carcass with feeding | vs | 30 h *Necrophila japonica* | 2.281344 | 11.619 | <0.001 |

**Table S5** Results of all possible pairwise comparisons of the concentration of dimethyl trisulfide (DMTS) based on the Tukey–Kramer test.

| Condition 1 |  | Condition 2 | SE | t-value | P-value |
| --- | --- | --- | --- | --- | --- |
| 12 h carcass with feeding | vs | 30 h rat carcass | 1.6429 | 0.547 | 0.9447 |
| 12 h carcass with feeding | vs | 30 h damaged carcass | 1.6429 | 1.994 | 0.2660 |
| 12 h carcass with feeding | vs | 30 h carcass with feeding | 1.6429 | 7.151 | <0.001 |
| 30 h rat carcass | vs | 30 h damaged carcass | 1.6429 | -1.447 | 0.5077 |
| 30 h rat carcass | vs | 30 h carcass with feeding | 1.6429 | 6.604 | <0.001 |
| 30 h damaged carcass | vs | 30 h carcass with feeding | 1.6429 | 5.157 | 0.0038 |
